# The application of positive psychology in the practice of education

**DOI:** 10.1186/2193-1801-3-147

**Published:** 2014-03-19

**Authors:** Anna Pluskota

**Affiliations:** Instutute of Sociology, Nicolaus Copernicus University, Fosa Staromiejska 1a, Toruń, 87-100 Poland

## Abstract

The purpose of the study is to present the possibility of the application in the field of education this highly interesting and promising trend in the psychology which is the positive psychology. For this reason the origins as well as an outline of the interest scope of this relatively recent, dating back only over 10 years, trend are shown. A crucial question has become in this context the examination of the relationship between the positive psychology and the education, particularly regarding the potential linked to the prospect of the so called strong points”. The founding fathers as well as the supporters of the positive psychology try to propagate it in the therapy and in the fields of organization and education. In the following text some selected concepts of the positive psychology and the corresponding examples of their practical use in the form of the so called positive prevention and intervention programs in the domain of education are described.

## Introduction

To begin with, some short explanation may be helpful. Despite the heading, this paper does not relate to what is or has been positive in education. The reason is not at all due to the fact that it is much easier to discuss the negative things about education, the diagnosis and analysis of problematical shortcomings and deficiencies, than it is to discuss positive aspects. Rather, the purpose of the article is simply to show the possibility of applying a new scientific concept to education – the concept of positive psychology. But before continuing, the difficulties of discussing positive aspects should be clarified.

Inspiration was given by a survey of scientific publications about psychology, covering both positive aspects and negative aspects. The survey had been conducted by a number of psychologists, including positive psychologists^a^, and it was decided to adopt their procedures for this paper. The database EBSCO was surveyed, covering a ten-year span. It was found that “education” was quite often correlated with negative subject matter. But this does not show that the database is unusual in any respect. It is surmised that surveys of other databases would show a similar result, and not only for education. It seems that sociology researchers are inclined in a specific way by their profession to penetrate all kinds of pathology and negative phenomena.

The same is true of the 20th Century literature of psychology. David G Myers draws attention to the fact that in “PsycInfo”, the electronic database of “Psychological Extracts”, for every twenty articles discussing negative emotions like prejudice, anxiety, anger, aggression, depression, etc., there is only one which deals with positive emotions like joy, satisfaction, happiness etc. (Myers 2000). Martin E P Seligman reports that for every hundred articles about sadness, there can be found only one about happiness. Contemporary psychology is preoccupied with the negative side of life, and interprets the functioning of the person only in terms of a disease model, ignoring almost completely the positive side of life (Seligman and Csikszentmihalyi 2000; Seligman 2005a). In the opinion of Seligman, contemporary psychology comes close to being “victimology”, a science of victims and injuries^b^.

Moreover, he believes that the social sciences have become sciences of “isms” (e.g. racism, sexism, ageism) and of all kinds of pathologies. They now exclude responsibility, the ability to make decisions, and free will, and consider individuls to act like puppets on strings, being controlled by their race, class, sex and gender roles and by “incentives” arising from their environment (Seligman 2005a).

Positive psychology has also emerged in specific reaction to the deteriorating social indicators in many economically and socially developed countries, because the progress of their economies, the growing wealth of states and their citizens, is not matched by an increase in life satisfaction, i.e. to mental well-being.

According to the World Health Organization, mental depression is the main cause of disability, and had become by the year 2000 the fourth gravest health problem throughout the world (see the YLD-indicator (**Y**ears of life **L**ost due to **D**isability) and the DALY- indicator (**D**isability-**A**djusted **L**ife **Y**ears) in the website of the World Health Organization (Mental Health, 2001). WHO experts estimate that by 2020 depression will have become the second largest health problem in the world. Also, the foremost proponents of positive psychology (among others Seligman 2005aMyers 2000) expect an epidemic of depression which will be particularly dangerous in view of the fact that depression increasingly affects younger people. According to the DALY indicator, in the age group 15–44 years depression is now the second greatest cause of disability, while suicide is one of the most frequent causes of death across the world, especially for young people (WHO official website, 2010).

An epidemic of depression, pessimism and lowered self-esteem in the young generation constitutes not only one of the major threats to mental health, but also becomes a serious social and economic problem Although positive psychology was conceived in reaction to deteriorating social indicators, that does not mean it is the only remedy. In the view of the founder of positive psychology, Martin Seligman, education is the most important weapon in combating and preventing the above identified problems and threats. Seligman attributed that particular significance to education from the very beginning, that is, since positive psychology was proclaimed. (It can be taken that positive psychology dates from his inauguration speech when he was elected President of the American Psychology Association in 1998). Hence, he argues that positive psychology alone is not capable of coping effectively with an epidemic of depression in the young generation; whereas education can be the best help, if based on the findings and solutions developed in the framework of the trend of thought described hereunder. So, what can the “new” positive education add to social practice and theory? In order to answer this question, it is necessary to mention, at least in a nutshell, what positive psychology is and what it is not.

### Positive psychology – How it came about, and what its interests are

After World War II, psychology was concerned with only one theme – mental diseases and psychological problems, and their treatment or prevention. Hence, as already described, the functioning person was analysed almost exclusively in terms of a disease model, a model of deficits. Devoting so much attention to every kind of pathology and phobia resulted in the elimination of the very idea of a fulfilled individual, prospering community from psychological research. It also ignored or denied those possibilities and potentials which could be realised through accessing underlying basic strengths, (in a person, community or institution). As Seligman says, the support and regeneration of existing strengths is the most effective weapon in the therapeutic arsenal (Seligman [Bibr CR18][Bibr CR19]).

The objective of positive psychology is therefore to initiate a change in psychology as well as in the social sciences, a change to cause a re-orientation and turning away from being exclusively busy with repairing the worst things in life, towards developing the best qualities in life (Seligman [Bibr CR18][Bibr CR19]). Positive psychology as a science is based on three pillars The first is a positive life experience for individuals – **exploiting positive emotions**. The second pillar is a person’s positive physical properties – **exploiting positive personality traits**, mainly virtues and strengths, but also aptitudes. The third pillar is a positive society **– exploiting positive social institutions,** in particular those such as democracy, a strong family, and education which promotes positive development (Seligman 2005a). An eminent task of positive psychology is to provide a theoretical basis as well as practical solutions to enable people to improve their mental well-being and to achieve better physical health. This new trend focuses on scientific research into resources, strengths and happiness. It concentrates on understanding, explaining, and supporting happiness and well-being, as well as upon uncovering the factors influencing such states (Carr 2009Gable and Haidt 2005). Even though the name of the trend was chosen in order to emphasize its area of interest (i.e. what is positive) and to underscore its difference in relation to the post-war achievements in psychology (with their main focus on what is negative), positive psychology is not in itself a separate science in competition with the earlier trends in psychology. Seligman has himself stressed many times that positive psychology is not to be seen in terms of a paradigm shift, and that no dichotomization has occurred in the field of psychology. He is, rather, convinced that it would be a mistake to try to reduce the idea to the mere assumption that once positive psychology had been identified, all that is outside its area of interest would belong to a different type of “negative” psychology^c^.

Although exponents of positive psychology do not explain the functioning of person by the deficit model (explaining function in terms of making up for shortages, minimising pain, compensating for deficits, repairing damage) they do not deny the existence of shortages, deficits and suffering. They argue, however, in favour of the so-called positive model, or strength signatures model, which aims not merely to help the individual to return to normality (normality being understood to be an absence of disturbances), but above all to strive for optimal functioning and development. Positive psychologists are convinced that concentrating on the positive model expands the resources of individuals and of society, contributing to their flourishing and thus reducing the need for “traditional” psychological and social interventions. Also, they believe that the positive model is not in competition with, but complementary to, the deficit model.

Positive psychology should not be seen as research in opposition to the earlier psychology. In creating its new and original theoretical models, positive psychology uses – both theoretically and methodologically – the same set of tools as traditional psychology does. In view of this fact, there is no need for positive psychology to be built entirely as a new construct. There is only required “a change of the object of interest – just moving away from repairing what is worst in life towards creating what is best in life” (Seligman 2005a). It constitutes a necessary complement to traditional psychology, bringing in a more systematic and penetrating approach to studying and supporting the optimal functioning of human beings (Seligman et al. 2004).

Positive psychology is sometimes judged to be very close to the popular and pseudo-scientific trend of Positive Thinking. It is even sometimes identified with positive thinking^d^. This demonstrates once again how deceptive the term positive psychology can be. In fact, positive thinking (which is even sometimes regarded as a trend or sub-discipline of science) assumes that it is enough only to think positively in order automatically to be successful, be happy, rich, to enjoy good health and to feel good, and actually contradicts the findings of positive psychology. Positive thinking as a pop-psychological trend, method, or even ideology, but based on ignorance and lack of knowledge, is inefficient and deceptive. According to the positive psychologists (but not only positive psychologists), positive thinking is noxious.

### Positivity in education – a little about the relationship between positive psychology and education

Returning to the previously formulated question about the relationship between positive psychology and education, the connection between the former and educational psychology should be considered. Educational psychology deals – generally speaking, both in its theory and practice – with the development of the affective, cognitive and social competence of young people. Its basic tenets are anchored in humanistic psychology, in which positive psychology resembles it. The theoretical as well as the practical solutions of educational psychology are based on the assumption that research into the development of a young individual is to be made in the context of the impacts and requirements of social surroundings such as family, school setting and culture. The aim of educational psychology is to help young people find their self-esteem, the meaning of life, and to gain self-confidence. In this dimension, positive psychology converges with the assumptions and practice of educational psychology. The scope of the trend presented in this article is, however, wider when compared with educational psychology, which focuses merely upon some aspects of the quality of life; that is, those which are relevant to children and youths. The objective of positive psychology is to establish original theoretical models concerning the good life in general, with their practical large–scale application going beyond pre-adult educational settings. Hence, there is the possibility of practical implementation of positive psychology in a range of contexts related to a wider concept of education – that of lifelong learning.

Martin E P Seligman has ascribed – from the very beginning when positive psychology was called into being – an immense importance to education in the widest sense. Optimal functioning, improved mental well-being, and effective prevention of an epidemic of depression will not be possible unless the concepts of hard determinism are questioned; the hard determinism which treats the individual as a victim of his or her own biological and socio-demographic characteristics – genes, class, race, gender, material wealth, etc.- and as a prisoner of his or her own past^e^.

The consequence of such hard determinism, so particularly characteristic of the social sciences (especially sociology, psychology and pedagogy), is the widespread view that the past determines both the present and the future, and leads to over-emphasis on negative events and negative emotions. Seligman believes that pessimistic assumptions about the future obstruct or even prevent proper development. They are completely unhelpful whenever development is at stake; in contrast, positive assumptions about the future precede and facilitate progress.

Seligman suggests that the field of education, particularly as far as the younger generation is concerned, should turn to look to the future, should focus on positive emotions, social commitment, the search for meaning, for harmony in human relations; on positive achievements, volition, and freedom, as well as upon health and growth^f^. Positive psychology postulates an understanding of individuals as being neither restricted nor pre-determined, as having personal will and freedom, possessing the potential for growth arising from her or his own **strength signatures and virtues.** Accordingly, says Seligman, positive psychology can be useful in education and become a reliable tool for definite increases in mental well-being. Even convinced as he is that positive psychology should be present in education, he asks whether mental well-being is something to be learned; whether it is – if it can be put so – learnable. He answers this question affirmatively. In order to support this assertion, he refers to empirically validated evidence obtained from practical applications of positive psychology, which shows that it is. Among the available evidence there are programs of so-called positive intervention in the field of education, such as for example the training of optimism – the Penn Resiliency Program – or supporting positive emotions – Three Blessings – or the diagnosis and further development of strengths – Signature Strengths^g^.

### Programs of positive intervention – some examples of the application of positive psychology in the field of education

#### Optimism

The Penn Resiliency Program (PRP) is a program designed to incorporate, as its main message, prevention of and resistance against the depression epidemic among youngsters^h^. It is based upon the psychological concept stating the highly consequential fact that our convictions regarding events and their interpretation have an impact not only on our emotions but also on our behavioural patterns. The theoretical foundation of this program is the concept of optimism-pessimism elaborated by Seligman, in which optimism and pessimism, considered as relatively durable human traits, have a decisive impact on the total functioning of human beings. They generate activity or passivity, and determine motivation and the choice of strategy for action, and the shape as well as the ways in which life objectives are realized. Optimism or pessimism account for the ability to transcend one’s own limits, or for being overwhelmed by feelings of helplessness (Pluskota-Lewandowska 2000,2009). They are both learned predispositions, despite their relatively permanent character. Therefore the PRP – leaning on the assumptions of cognitive-behavioural therapy – adopts a wide range of methods and tools to induce an optimistic style of personal development^i^. Positive intervention of the kind exemplified by the PRP has its own empirical foundation, its effects having been evaluated through longitudinal studies conducted by various research teams in the different continents where the PRP has been implemented. As pointed out by the researchers, the program has proved to be highly effective in reducing the indicators of depression, as measured by the use of standardized scales of helplessness, hopelessness, and depression. The results of the longitudinal studies show that this effect is still maintained two years after the completion of the program. Researchers are convinced that the empirical data proves the long-lasting positive effects of the intervention (Cutuli et al. 2006Gillham et al. 1995Gillham and Reivich 1999Roberts et al. 2004Seligman et al. 2005).

The measurements carried out a short time after ending the PRP, and the measurements made after six months or even after two years, provide the evidence that a learned optimistic attitude to success and failure is used decidedly more often among young people who took part in the project, than in the control group who had not taken part. Over the past 20 years, two thousand children between the ages of 8 and 15 years, and from different countries, have participated in the program. The empirically validated effects, as demonstrated by the evaluations, show an increase in psychological resilience, prevention and reduction of the symptoms of depression, the minimising of feelings of hopelessness, lower levels of clinical depression, the prevention or reduction of anxiety, and reduced aggression and criminality (Seligman et al. 2009).

#### Social commitment – signature strengths

Diagnosing and further developing strengths – this is the proposal widely promoted by Martin E P Seligman, based on the concept of so-called signature strengths. It is interesting to note that this concept emerged to meet needs in the field of education, notwithstanding the fact that as its authors and supporters believe, it can be more widely applied (signature strengths being, in practice, applicable for any individual, independently of that person’s social and demographic characteristics). The immediate reason, the driving force leading to this conception, was the necessity of constructing an effective intervention program for young people, financed by the Education department of the United States of America. A representative of that department asked Martin Seligman for help in 1999. He in turn convoked and assembled a team of scholars, presided over by Christopher Peterson. The main idea of the team was the assumption that any intervention aiming to improve the “character” of the youths would not be possible until those in charge of the program had acquired a background knowledge of what was to be made better. This is how the idea of a “taxonomy of good character” arose – the classification of strengths (Seligman 2005a). In consequence, the main task for the scholars and practitioners involved in the reported projects (and consequently a task for positive psychology) became to identify and elaborate a classification of the resources, strengths and virtues of a person. This would offer a counterpart to the psycho-pathological classification as presented in the Diagnostic and Statistical Manual of Mental Disorders (DSM). The result submitted by the team led by Christopher Peterson is a formal list of character strengths and virtues, abbreviated to “CSV”, which was presented in a detailed form in the book published by Peterson and Seligman 2004, titled “Character Strengths and Virtues: a Handbook and Classification (Peterson and Seligman 2004). As mentioned above, the adherents of this classification believe that it is exhaustive and universally valid because it represents values which are recognised and held in high esteem across all known cultures. The classification embraces six virtues: wisdom, courage, humanity, justice, temperance, and transcendence. These six virtues correspond with 24 character strengths composing various individualized structures (hence the name of “signature strengths”), which characterize and spell out the individual traits of each person (Seligman 2005a). The intervention of positive psychology, but based upon the concept of signature strengths, differs, according to Seligman, from many prevention and healing procedures in psychology (positive psychology included) inasmuch as it makes use and takes advantage of an individual’s inherent personal resources and strengths. The point is to develop virtues and signature strengths by identifying and harnessing them (Seligman 2005a). Furthermore, Seligman argues that an individual should not use too much effort to reduce or eliminate weaknesses, because success in life and emotional satisfaction stem from building and taking advantage of signature strengths, which should therefore be concentrated on. The respective virtues and corresponding strengths should be further developed and increased through selection as well as by undertaking tasks and challenges which consolidate those virtues and strengths. Seligman affirms that this is an additional and important guideline that can be successfully applied in the field of education – in teaching and prevention programs etc.

#### Positive emotions – “Three blessings”

Another kind of positive intervention is the “Three Blessings” program for supporting positive emotions. Its theoretical background is the theory of the broaden-and-build function of positive emotions formulated and advanced by Barbara Fredrickson. She succeeded in overcoming the traditional stereotype of thinking that well-being is a result of indicator of prosperity in life. Her argument is that positive emotion is not only a signal of well-being, but also one of the factors determining it, the feeling of happiness being not only an effect of prosperity in life, but also (and perhaps primarily) the basis for prosperity (Fredrickson 1998,2001). The theory of the broaden-and-build function of positive emotions is one of the empirically best-proven concepts in positive psychology. The results of experimental and longitudinal studies support Fredrickson in maintaining that positive affective experiences do not merely signal well-being, but also contribute to growth and development (Carr 2009) Positive emotions temporarily extend the repertoire of thoughts and patterns of behaviour, and this extension provides the chance for durable personal resources to be accumulated. They in turn enable individual development and transformation by creating positive or adaptive spirals of emotions, cognitive acts and actions. Enrichment of personal resources diminishes vulnerability and as a consequence increases the chances of experiencing even more positive emotions. Fredrickson calls this mechanism the upward spiral of positive emotions (Fredrickson 2003).

What can be the meaning of the theory of broaden-and-build action of positive emotions for education? The results of research into the education of children show that children in a positive frame of mind learn faster. Thanks to positive emotions, their intellectual resources (span of attention, creativity, and intuition) are increased, their social skills (like using or sharing the perspective of others, cooperation, and prosocial attitudes) rise, and their *physical fitnesss* improves (Fredrickson 1998). Positive emotions open up new opportunities and outlooks, fostering the extension of one’s range of vision to go beyond the repertoire of tested and habitual thoughts and activities. Overall, the indirect effect of positive emotions is an increase in the resources available to cope with stress. The active intervention project named Three Blessings was conceived as a simple exercise aimed at arousing positive emotions in children by way of a special examination of what had happened in the course of the previous day. The child is expected to recall three things which had happened (the importance of the things not needing to be significant) and which had gone successfully, “smoothly”, in that child’s opinion. Then the child follows with an explanation and interpretation of the causes for success. Those who are in favour of this kind of exercise point to its good effect, validated by empirical evidence, consisting of an enhanced feeling of happiness lasting for up to six months (Wallis 2005).

## Summary

Positive Psychology enjoys, as hardly any of the new trends in psychology ever did before, a high degree of popularity and spontaneous recognition among numerous scholars and outsiders alike. It is a brilliant example of well-selling (both figuratively and literally) scientific knowledge with multiple aspects: therapeutic (in various forms of therapy), educational, in human resource management, in organizational management, and so on. There are many examples which demonstrate that the findings of positive psychology can be applied practically beyond the immediate domain of psychology. However, this relatively fresh trend of thought is not completely without controversy. It is outside the scope of this article to quote and discuss the criticism attracted by positive psychology. Nevertheless, it should be noted that most of it is constructive.

The rising indicators of depression among young people throughout the world, and their low levels of life satisfaction, referred to even as a “depression epidemic”, are for positive psychologists a strong argument in favour of implementing the presented developments in the field of education. Positive education is defined by those psychologists both as an education which improves the teaching of traditional subjects, and as an education for happiness also. They consequently maintain that such attributes as psychological resilience, social commitment, and a sense of meaningfulness in life, should be transmitted to children by teaching at school. This would serve as a vehicle to increase satisfaction with life, to help children to learn, and to support them in their own development of creative thinking. School can become a place to enable young people to achieve large-scale development, and increase their personal resources and their mental well-being. School seems to be a perfect place for implementing positive initiatives (prevention and positive therapy) for learning mental well-being – most young people attend schools and spend most of their time at school (even in countries where attendance at elementary school is not obligatory).

### Websites

Authentic Happiness – the homepage of Dr. Martin Seligman, Director of the Positive Psychology Center at the University of Pennsylvania. http://www.authentichappiness.sas.upenn.edu/Default.aspx. Accessed 15 February 2014.

Official website of the Positive Psychology Center at the University of Pennsylvania. http://www.ppc.sas.upenn.edu/prplessons.pdf. Accessed 01 February 2014.

Official website of the University of Social Sciences and Humanities, http://www.swps.pl/uczelnia-wroclaw/seminarium-seligman/dyskusje-panelowe. Accessed 14 December 2010.

Official website of the World Health Organization. http://www.who.un.org.pl/aktualnosci.php?news=330&wid=9.pdf. Accessed 14 December 2010.

## Endnotes

^a^Among others Czapiński 2005Myers 2000Seligman 2005a.

^b^Despite the fact that positive psychology has been developing fast and became popular among psychologists it is not well known outside of the field. Therefore the goal of this article is to introduce the positive psychology to the readers that are not experts in the field or are not familiar with this trend at all. Consequently the introduction includes the origin of positive psychology using primarily Seligman’s notion of “Positive Psychology”. This is due to the fact that Seligman is recognized as one of the founding father of positive psychology who played the crucial role in creating theoretical pillars of the trend. He was also the one who announced the new trend to the world. Moreover the two out of three examples of positive psychological interventions in the education were based on Seligman’s concepts. The selection of the examples itself was not random – they have been empirically documented to the great extent and extensively discussed. They are also applied the most often in the education field.

^c^It should be noted however that the confrontational tone of early publications by Seligman – early in terms of the overall history of positive psychology – can suggest such a division.

^d^This statement is based on the author’s own experience in teaching “positive psychology” course at the Nicolaus Copernicus University, Toruń, Poland. Social science students often fail to recognize the difference between the “positive psychology” and “positive thinking” trend. It is also important to point out that the founders of Positive Psychology Center (PPC) at the University of Pennsylvania recognized this confusion among the Internet users interested in “positive psychology” and explained that “Positive psychology is different from positive thinking in three significant ways. First, positive psychology is grounded in empirical and replicable scientific study. Second, positive thinking urges positivity on us for all times and places, but positive psychology does not. Positive psychology recognizes that in spite of the advantages of positive thinking, there are times when negative or realistic thinking is appropriate.” [http://www.ppc.sas.upenn.edu/faqs.htm]. Kendra Cherry who specializes in making psychology more understandable for students, uses a similar approach. She is the author of the *Everything Psychology Book* (Cherry 2010). Despite the fact that the literature and the Internet resources on the subject include many similar clarifications, there are still many instances of mistaking “positive psychology” for “positive thinking” trend.

^e^Seligman speaks in this context about the intellectual legacy of Darwinism, Marxism, Freudianism, all of which he describes as dogmas (Intellectual Dogma Background) which treat the individual as a slave of genes and class, as a wage slave, or as a slave of one’s own psychic past related to sexuality and aggression (Seligman 2010).

^f^Quoted from the presentation held by Seligman during the Symposium Positive Psychology with Prof. dr. Seligman, Seligman Europe 2010, Wroclaw, Poland.; 3rd of July 2010 (Seligman 2010).

^g^More information on the subject of those programs can be found in the website pages of the Positive Psychology Center – http://www.ppc.sas.upenn.edu/index.html, in the pages of “Authentic Happiness” http://www.authentichappiness.sas.upenn.edu/Default.asnx as well as in the publications: Cutuli et al. 2006Gillham et al. 1995Gillham and Reivich 1999Jaycox et al. 1994Roberts et al. 2004Seligman et al. 2005Seligman et al. 2009Shimai et al. 2006, Zubernis et al. 1999.

^h^Seligman reports replications of the program to involve 3000 children before 2010 (Seligman 2010).

^i^Among those methods, tools, and techniques are the following forms: role-playing, short stories, animated cartoons, all of which provide the possibility of gaining an understanding of the point of the above-noted concept. Suitable for this purpose could also be group discussion of the use of the learned skills in hypothetical as well as real situations.
